# The relationship between MHC-*DRB*1 gene second exon polymorphism and hydatidosis resistance of Chinese merino (Sinkiang Junken type), Kazakh and Duolang sheep

**DOI:** 10.1051/parasite/2011182163

**Published:** 2011-05-15

**Authors:** R.Y. Li, W.Q. Hui, B. Jia, G.Q. Shi, Z.S. Zhao, H. Shen, Q. Peng, L.M. Lv, Q.W. Zhou, H.T. Li

**Affiliations:** 1 College of Animal Science and Technology, Shihezi University Shihezi Sinkiang 832003 P. R. China

**Keywords:** Chinese merino sheep (Sinkiang Junken type), Kazakh sheep, Duolang sheep, Ovar-*DRB*1 exon 2, PCR-RFLP, hydatidosis, mouton mérinos chinois (type Sinkiang Junken), mouton Kazakh, mouton Duolang, Ovar-DRB1 exon 2, PCR-RFLP, hydatidose

## Abstract

The present study aimed at detecting the association of ovine major histocompatibility complex class II (Ovar II) *DRB*1 gene second exon and susceptibility or resistance to hydatidosis in three sheep breeds of Sinkiang. The MHC-*DRB*1 second exon was amplified by polymerase chain reaction (PCR) from DNA samples of healthy sheep and sheep with hydatidosis. PCR products were characterized by the restriction fragment length polymorphism (RFLP) technique. Five restriction enzymes, *Mva*I, *Hae*III, *Sac*I, *Sac*II, *Hin*1I, were used, yielding 14 alleles and 31 restriction patterns. Frequencies of patterns *Mva*Ibc, *Hin*1Iab, *Sac*IIab, *Hae*IIIde, *Hae*IIIdf, *Hae*IIIdd (*P* < 0.01) in Kazakh sheep, *Sac*Iab (*P* < 0.05) in Duolang sheep, and *Hae*IIIab, *Hae*IIIce, *Hae*IIIde, *Hae*IIIee (*P* < 0.01) in Chinese Merino (Sinkiang Junken type) sheep, were significantly higher in healthy sheep compared with infected sheep. These results indicated a strong association between these patterns and hydatidosis resistance. In contrast, the frequencies of *Mva*Ibb, *Sac*IIaa, *Hin*1Ibb, *Hae*IIIef (*P* < 0.01) and *Hae*IIIab (*P* < 0.05) in Kazakh sheep, *Sac*Ibb, *Hae*IIIae, *Hin*1Iab (*P* < 0.05), *Hae*IIIaa, *Hae*IIIbe, *Hae*IIIef (*P* < 0.01) in Duolang sheep, *Sac*IIaa (*P* < 0.05) and *Hae*IIIbd, *Hin*1Ibb, *Hae*IIIcf, *Hae*IIIef (*P* < 0.01) in Chinese Merino sheep (Sinkiang Junken type) were significantly lower in healthy sheep compared with infected sheep. This indicated a strong association between these patterns and hydatidosis susceptibility. In addition, sheep with the pattern of *Hae*IIIef demonstrated a high hydatidosis susceptibility (*P* < 0.01) in all three breeds, while sheep with the pattern *Hae*IIIde demonstrated significant hydatidosis resistance (*P* < 0.01) in Kazakh and Chinese Merino sheep (Sinkiang Junken type). These results suggest that the Ovar-*DRB*1 gene plays a role in resistance to hydatidosis infection in the three sheep breeds.

## Abstract (fr)

Le but de cette étude était de rechercher les associations éventuelles entre le deuxième exon du gène *DRB*1 du complexe majeur d’histocompatibilité de classe II (Ovar II) et la susceptibilité ou la résistance à l’échinococcose chez trois races de moutons au Sinkiang. Le deuxième exon a donc été amplifié par PCR à partir d’échantillons d’ADN de moutons sains ou infectés. Les produits de PCR ont été caractérisés par la technique de polymorphisme de la longueur des fragments obtenus après digestion enzymatique. Cinq enzymes ont été utilisées : *Mva*I, *Hae*III, *Sac*I, *Sac*II, *Hin*1I, produisant 31 profils de restriction correspondant à 14 allèles. Chez les moutons sains, la présence de certains profils est significativement plus élevée que chez les moutons infectés. Il s’agit des profils *Mva*Ibc, *Hin*1Iab, *Sac*IIab, *Hae*IIIde, *Hae*IIIdf, *Hae*IIIdd (P < 0,01) chez les moutons Kazakh, de *Sac*Iab (*P* < 0,05) chez les moutons Duolang, et de *Hae*IIIab, *Hae*IIIce, *Hae*IIIde, *Hae*IIIee (*P* < 0,01) chez les moutons mérinos chinois (type Sinkiang Junken). Ces résultats indiquent une forte association entre ces profils particuliers et la résistance à l’échinococcose. Au contraire, la fréquence d’autres profils est significativement plus faible chez les animaux non infectés. Il s’agit de *Mva*Ibb, *Sac*IIaa, *Hin*1Ibb, *Hae*IIIef (*P* < 0,01) et *Hae*IIIab (*P* < 0.05) chez les moutons Kazakh, *Sac*Ibb, *Hae*IIIae, *Hin*1Iab (*P* < 0.05); *Hae*IIIaa, *Hae*IIIbe, *Hae*IIIef (*P* < 0,01) chez les moutons Duolang, *Sac*IIaa (*P* < 0,05) et *Hae*IIIbd, *Hin*1Ibb, *Hae*IIIcf, *Hae*IIIef (*P* < 0,01) chez les moutons mérinos chinois (type Sinkiang Junken). Ceci indique une forte association de ces profils avec la susceptibilité à l’échinococcose. De plus, une susceptibilité très importante à l’échinococcose a été observée pour le profil *Hae*IIIef chez les toutes les races de moutons (*P* < 0,01), alors que le profil *Hae*IIIde est associé à une résistance significativement plus élevée (*P* < 0,01) dans le cas des moutons Kazakh et des moutons mérinos chinois (type Sinkiang Junken). Ces résultats suggèrent que le gène Ovar-*DRB*1 joue un rôle dans la résistance à l’échinococcose chez les trois races de mouton.

## Introduction

Hydatidosis (*Echinococcus granulosus*) is recognized as one of the world major zoonoses, and is found all over the world (Rausch, 1995; Andersen *et al.*, 1997; Dalimi *et al.*, 2002; Eckert & Deplazes, 2004; Jenkins *et al.*, 2005). Sinkiang Autonomous Region of China is a prevalent area of hydatidosis. In sheep, the overall prevalence rate for hydatidosis cysts is 38.89% to 61.25% (Li *et al.*, 2005). Kazakh sheep and Duolang sheep, the local sheep of Sinkiang, are bred for meat and fat. Chinese Merino sheep (Sinkiang Junken type) produce excellent wool. In Sinkiang, hydatidosis in farm animals causes considerable economic problems due to the loss of meat and edible liver, as well as the value of the fleece from infected sheep. Therefore it also affects the life quality of herdsmen. In recent years, many animal breeding studies have focused on MHC genes as candidate genes for disease resistance and susceptibility. The MHC is a multigene family that controls immunological self/non-self recognition. They include genes for cell surface glycoproteins that present peptides of foreign and self proteins to T cells, thereby controlling both cell- and antibody-mediated immune responses (Klein, 1986). A striking characteristic of MHC genes is their extreme polymorphism.

Diversity driven by pathogens implies a strong association between MHC alleles and patterns of resistance to specific autoimmune or infectious diseases. Such a link was first shown for chickens, in which the B21 haplotype (MHC class IIB) confers the strongest resistance to the herpes virus responsible for Marek’s disease (Briles *et al.*, 1977; Longenecker & Gallatin, 1978). Equally well known is the role of the chicken class I MHC in providing resistance to the Rous sarcoma virus (Schierman & Collins, 1987; Kaufman & Venugopal, 1998). The polymorphism of Ovar-*DRB*1 plays an important role in resistance to nematode infection in the Suffolk breed (Sayers *et al.*, 2005).

MHC Class II Ovar-*DRB*1 was chosen as the immune response gene in this study because it is highly polymorphic, transcribed, and there are over 100 different *DRB*1 alleles reported in Genbank based upon either restriction fragment length polymorphisms (RFLP) or the deduced amino acid sequence for the β1 domain encoded by exon 2 (Dutia *et al.*, 1994; Schwaiger *et al.*, 1996; Jugo & Vicario, 2001; Konnai *et al.*, 2003; Herrmann *et al.*, 2005). Others have shown specific MHC-*DRB*1 alleles associate with resistance and/or less severe clinical signs in human hydatidosis (Gottstein *et al.*, 1996; Li *et al.*, 2003). Konnai *et al.* (2003) detected the Ovar-*DRB*1 exon 2 polymorphisms of Suffolk sheep by polymerase chain reaction restriction fragment length polymorphism (PCR-RFLP).

In the present study, the polymorphism of the class II Ovar-*DRB*1 exon 2 was detected by PCR-RFLP analysis in three sheep breeds. We characterized the relationship between the Ovar-*DRB*1 exon 2 polymorphism and hydatidosis resistance, and screened the genotypes associated with hydatidosis resistance and susceptibility in each sheep breed. Results of the present study may play an important role in developing new sheep breeds that are resistant to hydatidosis.

## Materials and Methods

### Animals sampling and sample preparation

Blood samples of Chinese Merino sheep (Sinkiang Junken type; 604 healthy animals and 425 animals with hydatidosis) were donated by agricultural construction division 9 in Sinkiang. Blood samples of Duolang sheep (122 healthy sheep and 70 sheep with hydatidosis) were donated by agricultural construction division 3. Blood samples of Kazakh sheep (400 healthy sheep and 302 sheep with hydatidosis) were donated by agricultural construction division 4. The sheep with hydatidosis were distinguished from healthy sheep by a commercially available enzyme-linked immunosorbent assay (ELISA) kit (Shenzhen Combined Biotech Co., Ltd, Shanghai, China). Genomic DNA were obtained from whole blood by phenol-chloroform method (Liu *et al.*, 1997), and stored in a - 20 °C freezer until analysis.

### Design of Ovar-*DRB*1 exon 2-specific primers and PCR amplification

The second exon of Ovar-*DRB*1 was amplified by PCR in two rounds. The first round of PCR was performed with primers OLA-ERB1 (5’-CCG GAA TTC CCG TCT CTG CAG CAC ATTTCT T-3’) and HL031 (5’-TTT AAA TTC GCG CTC ACCTCG CCG CT-3’) (adopted from Konnai *et al.*, 2003). We subjected 100 ng of genomic DNA to PCR amplification in a total volume of 20 μl, containing 1.5 mM MgCl_2_, 120 μM dNTP, 0.2 mM each primer, and 1.5 U of Taq polymerase (TIANGEN Biological Engineering Technology And Service Company, Beijing, China). Reactions were performed in a thermocycler (Bio RAD, Germany) under the following conditions: a single cycle of 5 min at 94 °C, followed by 15 cycles of 94 °C for 30 s, 50 °C for 30 s, and 72 °C for 60 s, with a final extension at 72 °C for 10 min. We used 3 μl of the resulting mixture and primers OLA-ERB1and OLA-XRBI (5’-AGC TCG AGC GCT GCA CAG TGAAAC TC-3’) (adopted from Konnai *et al.*, 2003) for the second round of PCR. The cycling conditions for the second round were: a single cycle of 5 min at 94 °C, followed by 30 cycles of 94 °C for 30 s, 63 °C for 30 s, and 72 °C for 60 s with a final extension at 72 °C for 10 min.

### Polymorphism detection by RFLP

PCR products (10 μl) from the second round were digested for 4 h at 37 °C with 5 U of *Mva*I, *Hae*III, *Sac*I, *Sac*II, or *Hin*1I (Shanghai Sangon Biological Engineering Technology And Service Co., Ltd., Shanghai, China) in a total volume of 20 μl. The products of enzyme digestion were analyzed by a 2.5% or 3% agarose gel electrophoresis.

### Statistical analysis

Hardy-Weinberg equilibrium of Ovar-*DRB*1 genotypes was analyzed by χ^2^ test. The distribution of genotypic frequency in healthy sheep and sheep with hydatidosis within a breed was analyzed by χ^2^ test. SPSS version 13.0 was used for statistical analysis.

## Results

### PCR amplification

**Fig 1.**
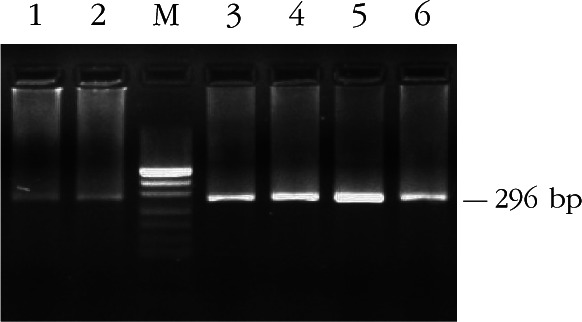
Electrophoretic patterns of PCR product of the second exon of Ovar-*DRB*1 in Kazakh sheep, M: PUC19 DNA marker

Ovar-*DRB*1 exon 2 was amplified by PCR with primers OLA-ERB1, OLA-HL031, and OLA-XRBI. A 296-bp band corresponding to the expected size of exon 2 was observed by 1.5% agarose gel electrophoresis (Fig. 1).

### PCR-RFLP

**Fig 2.**
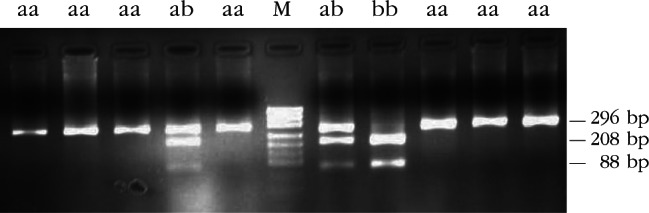
Electrophoretic patterns of the second exon of Ovar-*DRB*1 digested with *Sac*I in Kazakh sheep, M: puc19 DNA marker.

**Fig 3.**
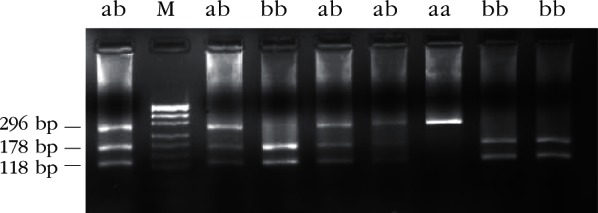
Electrophoretic patterns of the second exon of Ovar-*DRB*1 digested with *Hin*1I in Kazakh sheep, M: puc19 DNA marker.

**Fig 4.**
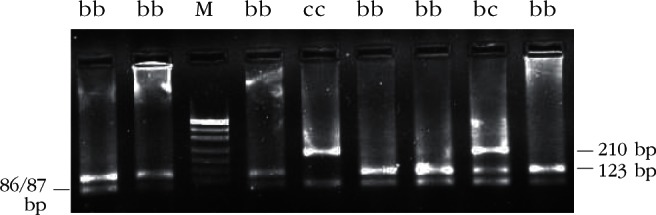
Electrophoretic patterns of the second exon of Ovar-*DRB*1 digested with *Mva*I in Kazakh sheep, M: puc 19DNA marker.

**Fig 5.**
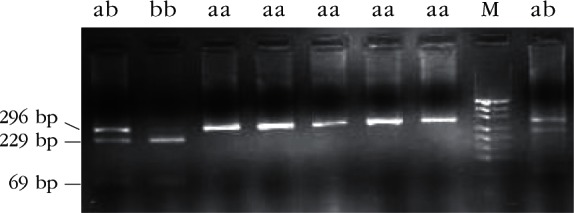
Electrophoretic patterns of the second exon of Ovar-*DRB*1 digested with *Sac*II in Kazakh sheep, M: puc19 DNA marker.

**Fig 6.**
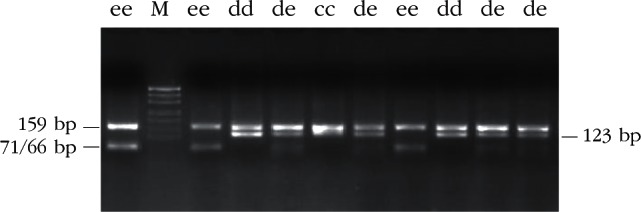
Electrophoretic patterns of the second exon of Ovar-*DRB*1 digested with *Hae*III in Kazakh sheep, M: puc19 DNA marker.

**Fig 7.**
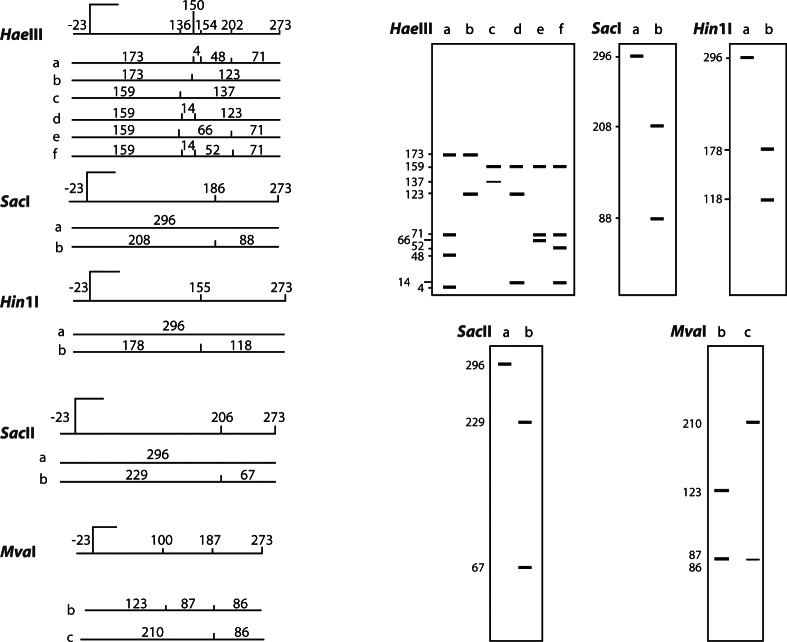
The diagram of the cleavage sites and fragments in MHC-*DRB*1 second exon.

Restriction enzyme analysis with *Sac*I, *Hin*1I, *Mva*I, *Sac*II and *Hae*III produced restriction patterns and allele frequencies in accordance with that reported by Konnai *et al.* (2003). Restriction patterns are shown in Table I (*Sac*I, *Hin*1I, *Mva*I, *Sac*II) and Table II (*Hae*III); fragments the genotypic restriction map is shown in Figs 2-6; and a diagram of this exonic region, the cleavage sites is shown in Fig. 7. The Ovar-*DRB*1 exon 2 of Chinese Merino, Duolang and Kazakh sheep was analyzed by PCR-RFLP using restriction enzymes *Sac*II (two alleles, three genotypes), *Mva*I (two alleles, three genotypes), *Sac*I (two alleles, three genotypes), *Hin*1I (two alleles, three genotypes), and *Hae*III (six restriction profiles, 19 patterns). Polymorphisms were detected at base pairs 229, 225, 208, 210, 178, 173, 159, 87.

**Table I.** PCR-RFLP genotypes of the second exon of the MHC-*DRB*1 gene.

Restriction enzymes		The genotypes of each restriction enzyme	
*Sac*I	aa (296bp)	ab (296bp/208bp/88bp)	bb (208bp/88bp)
*Hin*1I	aa (296bp)	ab (296bp/178bp/118bp)	bb (178bp/118bp)
*Mva*I	bb (123bp/87bp/86bp)	bc (210bp/123bp/87bp/86bp)	cc (210bp/86bp)
*Sac*II	aa (296bp)	ab (296bp/229bp/69bp)	bb (229bp/69bp)

**Table II.** The genotypes of PCR-RFLP by restriction enzyme *Hae*III in the second exon of the Ovar-*DRB*1 gene.

Genotypes	Restriction fragments (bp)	Genotypes	Restriction fragments (bp)
*Hae*III aa	173/71/48/4	*Hae*III af	173/159/71/52/48/14/4
*Hae*III bb	173/123	*Hae*III bd	173/159/123/14
*Hae*III cc	159/137	*Hae*III be	173/159/123/71/66
*Hae*III dd	159/123/14	*Hae*III cd	159/137/123/14
*Hae*III ee	159/71/66	*Hae*III ce	159/137/71/66
*Hae*III ff	159/71/52/14	*Hae*III cf	159/137/71/52/14
*Hae*III ab	173/123/71/48/4	*Hae*III de	159/123/71/66/14
*Hae*III ac	173/159/137/71/48/4	*Hae*III df	159/123/71/52/14
*Hae*III ad	173/159/123/71/48/4	*Hae*III ef	159/71/66/52/14
*Hae*III ae	173/159/71/66/48/4		

### CHI-square analysis

The Ovar-*DRB*1 exon 2 alleles of three breeds were analyzed by χ^2^ test to determine whether they were consistent with the Hardy-Weinberg distribution, using data shown in Table 3. The χ^2^ value of the patterns in Kazakh sheep were 173.85 (*Mva*I; 2 degrees of freedom [df]; *P* < 0.01), 9.24 (*Sac*I; 2 df; *P* < 0.01), 0.33 (*Sac*II; 2 df; *P* > 0.05), and 5.84 (*Hin*1I; 2 df; *P* > 0.05). These results indicated that patterns produced with restriction enzymes *Sac*II and *Hin*1I were in Hardy-Weinberg equilibrium, while patterns produced by restriction enzymes *Mva*I and *Sac*I were not.

The χ^2^ value of patterns in Duolang sheep were 1.25 (*Mva*I; *P* > 0.05), 13.63 (*Sac*I; *P* < 0.01), 7.44 (*Sac*II; *P* < 0.05), and 16.11 (*Hin*1I; *P* < 0.01). These results suggested that patterns produced by *Mva*I were in Hardy-Weinberg equilibrium, while patterns produced by *Sac*I, *Sac*II and *Hin*1I were not.

The χ^2^ value of patterns in Chinese Merino sheep were 0.03 (*Mva*I; *P* > 0.05), 1.62 (*Sac*I; *P* > 0.05), 0.38 (*Sac*II; *P* > 0.05), and 2.14 (*Hin*1I; *P* > 0.05). These results suggested that patterns produced by *Mva*I, *Sac*I, *Sac*II, and *Hin*1I were in Hardy-Weinberg equilibrium.

### Relationship between Ovar-*DRB*1 genotypes and hydatidosis resistance

Comparison of genotypes in sheep with hydatidosis and healthy controls is shown in Table III. Analysis revealed a higher frequency of patterns *Mva*Ibc, *Hin1*Iab, *Sac*IIab, *Hae*IIIde, *Hae*IIIdf, and *Hae*IIIdd (*P* < 0.01) in Kazakh sheep, *Sac*Iab (*P* < 0.05) in Duolang sheep, and *Hae*IIIab, *Hae*IIIce, *Hae*IIIde, and *Hae*IIIee (*P* < 0.01) in Chinese Merino sheep (Sinkiang Junken type) in healthy sheep compared with infected sheep, indicating a strong association between these patterns and hydatidosis resistance. Frequencies of patterns *Mva*Ibb, *Sac*IIaa, *Hin*1Ibb, *Hae*IIIef (*P* < 0.01) and *Hae*IIIab (*P* < 0.05) in Kazakh sheep, *Sac*Ibb, *Hae*IIIae, *Hin*1Iab (*P* < 0.05) and *Hae*IIIaa, *Hae*IIIbe, *Hae*IIIef (*P* < 0.01) in Duolang sheep, *Sac*IIaa (*P* < 0.05) and *Hae*IIIbd, *Hin*1Ibb, *Hae*IIIcf, *Hae*IIIef (*P* < 0.01) in Chinese Merino sheep (Sinkiang Junken type) were lower in healthy sheep compared with infected sheep, indicating a strong association between these patterns and hydatidosis susceptibility.

**Table III.** Genotypic frequencies of the second exon of the MHC-*DRB*1 gene in healthy group and hydatidosis group of three breeds sheep.

	Chinese Merino sheep (Sinkiang Junken type)		Kazakh sheep		Duolang sheep	
Genotypes	Controls	Hydatidosis Sheep	Controls	Hydatidosis Sheep	Controls	Hydatidosis Sheep
*Mva*Ibb	0.668 (392)	0.650 (262)	0.623 (246)	0.798** (241)	0.569 (62)	0.593 (32)
*Mva*Ibc	0.291 (171)	0.325 (131)	0.377** (149)	0.199 (60)	0.349 (38)	0.333 (18)
*Mva*Icc	0.041 (24)	0.025 (10)	0 (0)	0.003 (1)	0.083 (9)	0.074 (4)
*Sac*Iaa	0.472 (262)	0.495 (207)	0.546 (219)	0.481 (142)	0.312 (38)	0.386 (27)
*Sac*Iab	0.452 (251)	0.416 (174)	0.354 (142)	0.390 (115)	0.648* (79)	0.486 (34)
*Sac*Ibb	0.076 (42)	0.089 (37)	0.100 (40)	0.129 (38)	0.041 (5)	0.128* (9)
*Sac*IIaa	0.612 (322)	0.686** (277)	0.704 (285)	0.805** (243)	0.500 (43)	0.444 (20)
*Sac*IIab	0.338 (178)	0.280 (113)	0.277** (112)	0.185 (56)	0.454 (39)	0.511 (23)
*Sac*IIbb	0.049 (26)	0.035 (14)	0.020 (8)	0.010 (3)	0.047 (4)	0.045 (2)
*Hin*1Iaa	0.224 (124)	0.195 (82)	0.355 (142)	0.361 (109)	0.107 (13)	0.029 (2)
*Hin*1Iab	0.495 (274)	0.441 (185)	0.498** (199)	0.371 (112)	0.549 (67)	0.714* (50)
*Hin*1Ibb	0.282 (156)	0.364** (153)	0.148 (59)	0.268** (81)	0.344 (42)	0.257 (18)
*Hae*IIIaa	0.092 (50)	0.090 (38)	0.024 (10)	0.049 (15)	0.041 (5)	0.129** (9)
*Hae*IIIab	0.059** (32)	0.017 (7)	0.009 (4)	0.030* (9)	0.123 (15)	0.071 (5)
*Hae*IIIac	0.024 (13)	0.040 (17)	0.007 (3)	0.010 (3)	0.033 (4)	0.043 (3)
*Hae*IIIad	0 (0)	0.002 (1)	0.007 (3)	0.007 (2)	0.008 (1)	0 (0)
*Hae*IIIae	0.037 (20)	0.050 (21)	0.034 (14)	0.040 (12)	0 (0)	0.057** (4)
*Hae*IIIaf	0.009 (5)	0.019 (8)	0.010 (4)	0.013 (4)	0.049 (6)	0.028 (2)
*Hae*IIIbb	0.037 (20)	0.019 (8)	0 (0)	0 (0)	0.033 (4)	0 (0)
*Hae*IIIbd	0.004 (2)	0.021** (9)	0 (0)	0 (0)	0.025 (3)	0 (0)
*Hae*IIIbe	0.004 (2)	0.012 (5)	0 (0)	0 (0)	0.008 (1)	0.229** (16)
*Hae*IIIcc	0.092 (50)	0.126 (53)	0.092 (38)	0.113 (34)	0.074 (9)	0.057 (4)
*Hae*IIIcd	0.013 (7)	0 (0)	0 (0)	0 (0)	0 (0)	0 (0)
*Hae*IIIce	0.112** (61)	0.064 (27)	0.082 (34)	0.079 (24)	0.131 (16)	0.086 (6)
*Hae*IIIcf	0.013 (7)	0.066** (28)	0.034 (14)	0.013 (4)	0.098 (12)	0.014 (1)
*Hae*IIIdd	0.029 (16)	0.024 (10)	0.101** (42)	0.036 (11)	0.008 (1)	0 (0)
*Hae*IIIde	0.099** (54)	0.033 (14)	0.121** (50)	0.030 (9)	0.041 (5)	0 (0)
*Hae*IIIdf	0.037 (20)	0.042 (18)	0.053 (22)	0.013 (4)	0.057 (7)	0.057 (4)
*Hae*IIIee	0.220** (120)	0.149 (63)	0.253 (105)	0.318 (96)	0.148 (18)	0.071 (5)
*Hae*IIIef	0.073 (40)	0.173** (73)	0.113 (47)	0.192** (58)	0.008 (1)	0.114** (8)
*Hae*IIIff	0.050 (27)	0.052 (22)	0.058 (24)	0.056 (16)	0.115 (14)	0.043 (3)

The same genotypes in healthy group and hydatidosis group of one breed sheep,

* *P* < 0.05,

** *P* < 0.01.

## Discussion and Conclusion

At present, both domestic and international studies have indicated that MHC genes show extensive polymorphism in humans, mice, cattle (Blattman *et al.*, 1993; Xu *et al.*, 1993), sheep, goats (Amills & Francino, 1995, 1996; Yang *et al.*, 2006; Sun *et al*, 2004), and chickens (Xu *et al.*, 2005). Using PCR-RFLP, Yang *et al.* (2006) and Sun *et al,* (2004) investigated the MHC-*DRB*3 polymorphism in sheep and goats. Konnai *et al.* (2003) determined the Ovar- *DRB*1 exon 2 polymorphisms of 52 Suffolk sheep by PCR-RFLP with restriction enzymes *Sac*I (two alleles), *Sac*II (two alleles), *Hin*1I (two alleles), and *Hae*III (six alleles), which was consistent with our results. Peng *et al.* (2007) determined Ovar-*DRB*1 exon 2 polymorphisms of 211 Chinese Merino sheep (Sinkiang Junken type) by PCR-RFLP with restriction enzymes *Sac*I (two alleles, three genotypes) and *Hin*1I (two alleles, three genotypes), which was also consistent with our results. However, six alleles and 15 patterns were found using the restriction enzyme *Hae*III in their study, while six alleles and 19 patterns were found using this enzyme in the present study. This difference may be due to different numbers and species of sheep. Last but not the least, the genotypes of *Hae*III bc and bf weren’t appearance in the population which we chosen. Perhaps they will be found in a larger population.

Ovar-*DRB*1 is a principal member of MHC class II DRB in sheep (Deverson *et al.*, 1991; Scott *et al.*, 1991; Ballingall *et al.*, 1992). It is often used as a genetic marker in disease association studies (reviewed by Dukkipati *et al.*, 2006). Azab *et al.* (2004) demonstrated that people who carry human leukocyte antigen (HLA)-DR3 and HLA-DR11 were at high risk for cystic echinococcosis (CE), and those with HLA-DR3 were more susceptible to complications. Shcherbakov & Monje-Barredo (1989) showed that people with HLAB5 and B18 of HLA I antigen were at high risk for CE, while those with HLA-B14 and B27 had resistance to CE. In addition, Schwaiger *et al.* (1995) and Sayers *et al.*, (2005) found that MHC-*DRB*1 was related to nematode resistance. Taken together, these results show that MHC polymorphisms are closely associated with parasite resistance or susceptibility. In the present study, the Ovar-*DRB*1 exon 2 polymorphisms in three breeds of sheep were also shown to be associated with hydatidosis resistance and susceptibility (Table III).

Analysis of the restriction patterns revealed that Chinese Merino sheep (Sinkiang Junken type), Duolang sheep, and Kazakh sheep with the pattern *Hae*IIIef all had high hydatidosis susceptibility. Chinese Merino (Sinkiang Junken type) and Kazakh sheep with the pattern *Hae*IIIde had strong hydatidosis resistance; in Duolang sheep, the *Hae*IIIde appeared to confer some hydatidosis resistance, but the association was not statistically significant. In addition, restriction analysis using enzyme *Hae*III produced 19 patterns in Chinese Merino sheep (Sinkiang Junken type) in the present study; however, four of these patterns (*Hae*IIIbb, *Hae*IIIbd, *Hae*IIIbe, *Hae*IIIcd) were not detected in Kazakh sheep, and three of these patterns (*Hae*IIIcd, *Hae*IIIad, *Hae*IIIae) were not detected in Duolang sheep. Kazakh sheep are the local sheep of Sinkiang Province. The Duolang sheep is a cross between Feitun sheep from Afghanistan and local sheep from Kashi City of Sinkiang (Jiang *et al.*, 2006). The Chinese Merino sheep (Sinkiang Junken type) is a cross between a Merino ram from Australia and Sinkiang Junken sheep. The difference among the three breeds in *Hae*III patterns of the Ovar-*DRB*1 second exon may be due to different breeding histories.

In the present research, we screened restriction patterns associated with hydatidosis resistance and susceptibility in three sheep breeds by PCR-RFLP. Additional research is needed to determine whether these patterns could serve as genetic markers for hydatidosis.
